# Bilateral Change in Vertical Hoof Force Distribution in Horses with Unilateral Forelimb Lameness before and after Successful Diagnostic Anaesthesia

**DOI:** 10.3390/ani12182485

**Published:** 2022-09-19

**Authors:** Johanna R. Hoffmann, Florian Geburek, Jenny Hagen, Kathrin Büttner, Antonio M. Cruz, Michael Röcken

**Affiliations:** 1 Equine Clinic (Surgery, Orthopaedics), Faculty of Veterinary Medicine, Justus-Liebig-University Giessen, 35392 Giessen, Germany; 2 Clinic for Horses, University of Veterinary Medicine Hannover, Foundation, 30559 Hannover, Germany; 3 Institute of Veterinary Anatomy, Faculty of Veterinary Medicine, Leipzig University, 04103 Leipzig, Germany; 4 Unit for Biomathematics and Data Processing, Justus-Liebig-University Giessen, 35392 Giessen, Germany

**Keywords:** equine gait, stance phase, breakover, diagnostic anaesthesia, kinetics, Hoof^™^ System

## Abstract

**Simple Summary:**

Lameness is the most common cause of reduced performance in equids. Therefore, its detection, accurate diagnosis, and appropriate treatment are important for animal welfare and economics. Subjective evaluation of a lame horse by visual assessment is prone to error. To objectify the examination, several computer-based systems have been developed. While kinematic investigations focus on the detection of movement asymmetries (e.g., of head, pelvis, withers) with the help of position sensors, kinetic examinations are based on pressure measurement under the hooves. In the current study, horses with unilateral forelimb lameness were equipped with a non-invasive pressure measurement system on both forelimbs simultaneously. Bilateral vertical force distribution (in kg) was evaluated during all phases of stance (landing, midstance, breakover) before and after diagnostic anaesthesia. Vertical force was reduced on the lame limb compared to the sound limb before diagnostic anaesthesia. After positive diagnostic anaesthesia, asymmetries were neutralised: vertical force increased on the lame limb, with breakover being most affected. In conclusion, the current pressure measurement system can be used to objectify lameness examinations in a clinical setting. Both lameness and diagnostic anaesthesia influence the particular phases of stance differently. This might contribute to a better understanding of equine gait and lead to individually optimised shoes.

**Abstract:**

Kinetic examinations of horses with induced lameness as well as the effect of perineural anaesthesia in sound horses have shown promise, but clinical studies regarding the effect of diagnostic anaesthesia during the different stance phases are rare. Fourteen horses with unilateral forelimb lameness were examined with the Hoof™ System during trot to assess vertical force distribution (in kg) affecting both front hooves before and after diagnostic anaesthesia during landing, midstance, and breakover. For statistical analysis, a covariance analysis with repeated measurements regarding the limb (lame/sound) as well as anaesthesia (before/after) and the covariable body weight was performed. The *p-*values for the pairwise comparisons were adjusted using the Bonferroni–Holm correction (*p* < 0.05). For all phases of the stance, a significant interaction between the factors limb and anaesthesia was shown. Before diagnostic anaesthesia, vertical force was significantly reduced on the lame limb compared to the sound limb during landing (−25%, *p* < 0.001), midstance (−20%, *p* < 0.001) and breakover (−27%, *p* < 0.001). After anaesthesia, the difference between both forelimbs was not significant anymore for all phases. The vertical force on the lame limb increased significantly after positive anaesthesia during the whole stance phase, with breakover being most affected (+27%, *p* = 0.001). Pressure measurements with the Hoof™ System can be used to evaluate the effect of diagnostic anaesthesia in a clinical setting with pain-related vertical force asymmetries being neutralised after diagnostic anaesthesia. Breakover is the main event influenced by lameness.

## 1. Introduction

Visual evaluation of a lame horse before and after diagnostic anaesthesia is part of a standard lameness investigation. However, the objectivity of this method is limited, and it can often be an inaccurate assessment method [1,2,3,4,5].

To objectively quantify gait abnormalities, several inertial sensor-based and kinetic systems, such as pressure mapping-based systems, have been developed [6,7,8,9,10,11]. However, until now, kinetic evaluations have focussed on the effect of perineural anaesthesia in sound horses and those with experimentally induced lameness [12,13,14,15,16], while clinical studies are rare [17].

Pressure measurements may either be performed with systems embedded in the runway [10] or with foil-based systems fixed to the hooves. The latter provides the analysis of bilateral pressure distribution during clinical lameness examinations, as they enable the analysis of multiple, consecutive strides under various conditions without noticeable interference with the physiologic gait pattern of the horse [18,19]. Pressure distribution of the loading area during the different phases of the stance phase can be evaluated [20]. Previous studies showed that pressure distribution under the hoof in lame horses varies depending on the underlying pathology [21].

The objective of the present study was to determine the effect of diagnostic anaesthesia on bilateral vertical force distribution (in kg) in horses with unilateral forelimb lameness during all phases of the stance phase by using a non-invasive pressure measurement system (Tekscan Hoof System^®^, Tekscan^®^, Inc., South Boston, MA, United States) on both forelimbs simultaneously. 

The following hypotheses were tested:

1. Before diagnostic anaesthesia
  (a) Vertical force in kilograms on the lame limb is reduced compared to the sound limb during all parts of the stance phase (landing, midstance, breakover).
2. After positive diagnostic anaesthesia
  (a) Vertical force on the anaesthetised limb increases whereas the vertical force on the sound limb decreases, which leads to a more symmetrical bilateral vertical force distribution in kilograms during all parts of the stance phase (landing, midstance, breakover).
  (b) Following diagnostic anaesthesia, the maximum increase in vertical force on the lame limb occurs during midstance.

## 2. Materials and Methods

### 2.1. Horses

Data were acquired prospectively from 14 horses with unilateral forelimb lameness that were referred for lameness examination. After anamnesis and a general examination, a complete orthopaedic examination was performed by two independent experienced veterinarians, and the indication for diagnostic anaesthesia was confirmed. Lameness severity was divided into five degrees using a modified AAEP lameness score [3], using half units (e.g., 3.5) at the discretion of the examining clinician. Weight of the horses was estimated by measuring the chest circumference (Horse & Pony Weighing Tape, William Hunter Equestrian, Littlehampton, UK).

### 2.2. Data Collection

**Figure 1:**
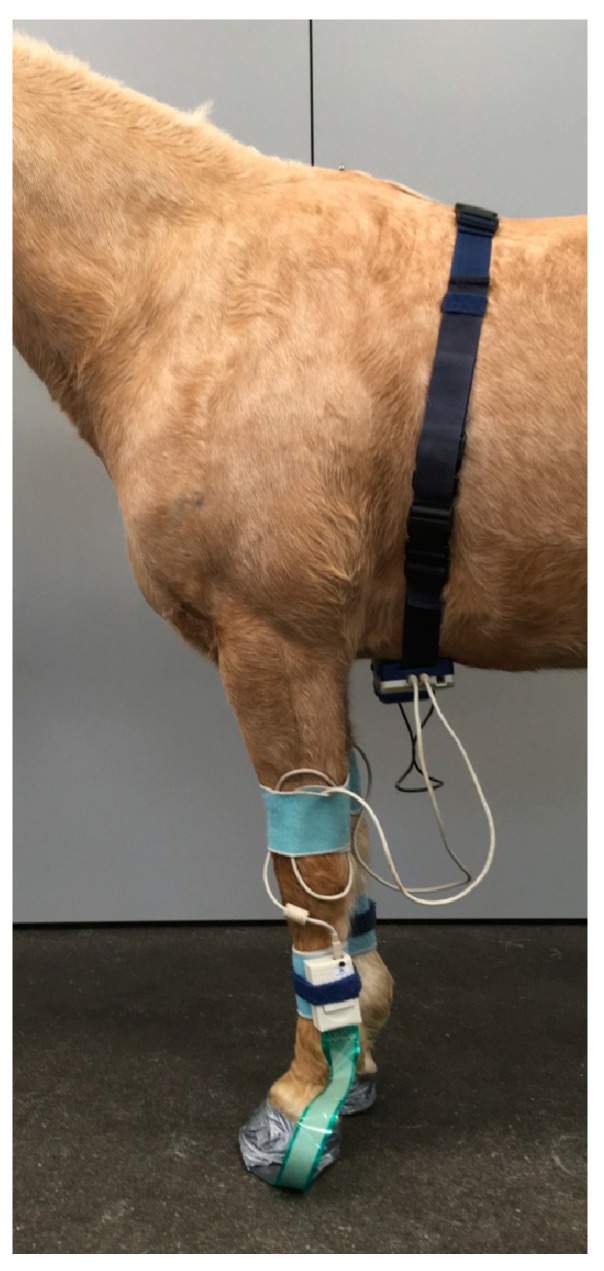
Horse equipped with the Hoof™ System.

**Figure 2:**
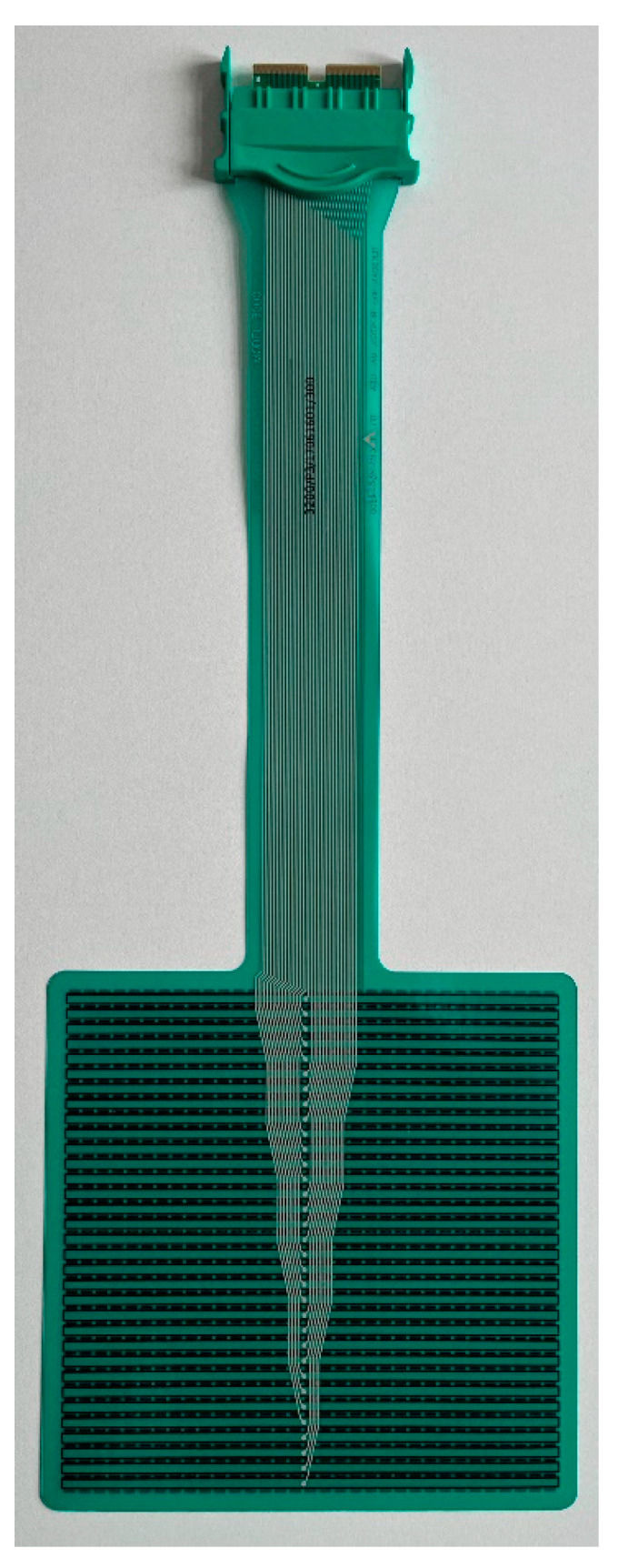
Sensor foil of the Hoof™ System.

**Figure 3:**
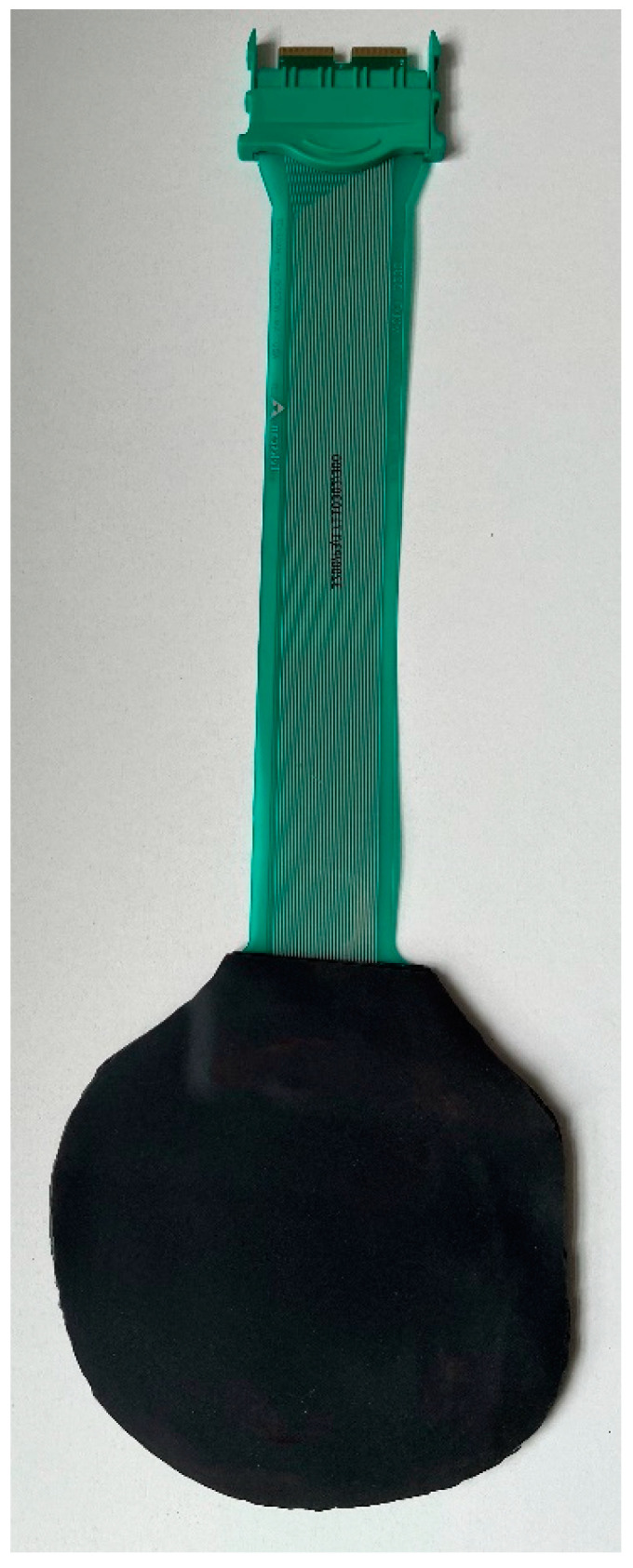
Prepared sensor foil.

All horses were examined with the Hoof™ System (TekScan^®^, TekScan Hoof System^®^) to assess vertical force (in kg) affecting both front hooves before and after diagnostic anaesthesia (Figure 1). The 0.15 mm thin sensor foils (Hoof Sensors Model #3200E, Tekscan, Inc., South Boston, United States) of the system detect vertical pressure with a spatial resolution of 3.9 sensor cells/cm² and a recording frequency of 250 Hz (Figure 2). After cutting the sensor foils to the respective size of the hooves they were protected on both sides with a self-adhesive 2 mm thick foam rubber layer (3M Deutschland GmbH, Neuss, Germany) (Figure 3) and fixed underneath both front hooves with adhesive tape (Tesa Duct Tape 4610, Global Headquarters—Tesa SE, Norderstedt, Germany). The connector of the sensor was inserted into the data logger on the lateral side of each forelimb. Before each measurement calibration was performed according to the manufacturers’ instructions and previous publications [18,22]. Subsequently, horses were trotted on hand at their natural speeds for 10 seconds in a straight line on a hard surface without sideway movements or excessive interaction with the leading person on the left-hand side of the horse while data were recorded and stored.

Afterwards, diagnostic anaesthesia of the affected leg was performed from distal to proximal by using defined volumes of 2% mepivacaine hydrochloride solution (Mecain^®^ 20 mg/mL: Puren Pharma GmbH & Co. KG, München, Germany) according to clinical guidelines [23,24]. Five to ten minutes post-injection, the effect of each regional nerve block was assessed by testing the skin sensitivity using a blunt item. Effectiveness of diagnostic intraarticular anaesthesia was assessed by obtaining a reflux of synovial fluid after needle placement into the synovial cavity and by clinical response.

Ten minutes after diagnostic anaesthesia, the horses were trotted again as described above to evaluate the subjective lameness degree. After successful anaesthesia, a second measurement with the Hoof™ System was performed. 

Examination protocols were standardised regarding the position of handler and timings following diagnostic anaesthesia. Horses were allowed to trot at their natural speed in a controlled environment ensuring regular movement during our investigations. Only strides from a regular segment were analysed, discarding the first and last strides to eliminate the effects of acceleration and deceleration.

### 2.3. Data Analysis

**Figure 4:**
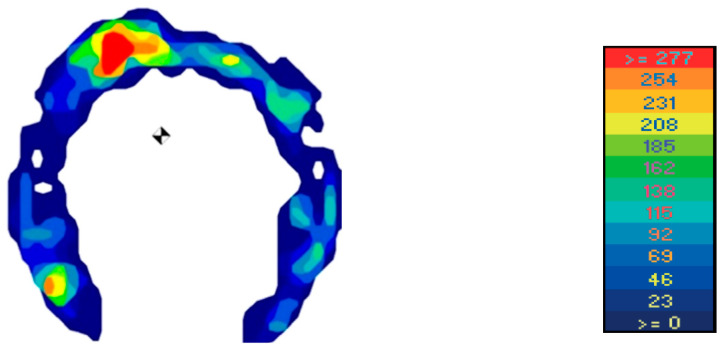
Averaged two-dimensional colour-coded pressure image during midstance. With a colour scale, the different pressure distributions (N/cm^2^) can be distinguished within the weight-bearing surface of the hoof (see pressure scale on the right side). In this study, data were converted to kilograms.

**Figure 5:**
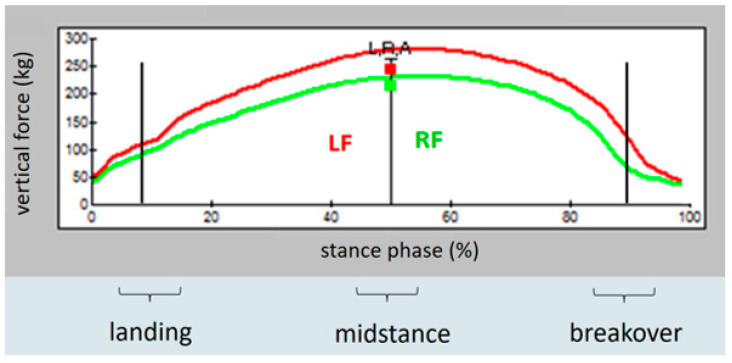
Averaged pressure–time curve of one horse in the current study with right forelimb lameness: vertical force in kg on the left (red) and the lame right (green) forelimb during landing (10% of the stance phase), midstance (50% of the stance phase), and breakover (90% of the stance phase) before anaesthesia.

**Figure 6:**
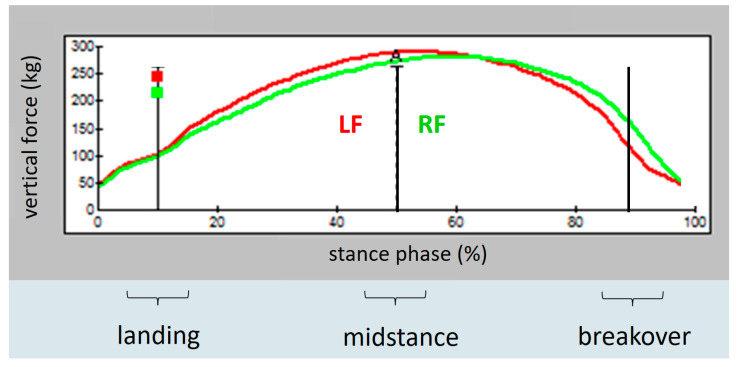
Averaged pressure-time-curve of one horse in the current study with right forelimb lameness: vertical force in kg on the left (red) and anaesthetised right (green) forelimb during landing (10% of the stance phase), midstance (50% of the stance phase), and breakover (90% of the stance phase) after anaesthesia.

After completed measurement, data were transferred to the software program FastSCAN Mobile Research Version 6.68^®^ (FastSCAN Mobile Research Version 6.68^®^ (Tekscan^®^), Inc., South Boston, MA, USA), displaying the information as averaged two-dimensional colour-coded pressure images (Figure 4) and as averaged pressure–time curves (Figure 5 and Figure 6). Generally, eight to ten consecutive strides were used for further calculations of the parameters of interest. 

The averaged pressure–time curves allowed for analysis of the vertical force distribution (in kg) at each single hoof and the difference between both forelimbs during landing, midstance, and breakover (Figure 5 and Figure 6). The x-axis of this curve shows the time of the whole stance phase. Approximately the first 20% of stance phase represents landing, followed by midstance, lasting until 80% of the time of stance phase. The last 20% represents breakover [25,26]. Representative points in time were used to assess vertical force exerted on the ground during landing (at the 10% point of stance phase in the pressure-time curves), midstance (at the 50% point of stance phase), and breakover (at the 90% point of stance phase) (Figure 5 and Figure 6).

For statistical analyses of the data, the software BMDP/Dynamic (BMDP Statistical Software Manual 1992: BMDP Release 8.1. University of California Press, Berkeley, CA, USA) was used. All variables were tested prior to the statistical analysis for normal distribution using the Shapiro–Wilk test. For each variable (vertical force in kilograms on both front hooves before and after diagnostic anaesthesia during landing, midstance, and breakover), a two-way analysis of covariance (ANCOVA) with repeated measurements regarding the factors limb (lame/sound) and anaesthesia (before/after) and the covariable body weight was performed. *p* < 0.05 was regarded as statistically significant. The *p-*values for the pairwise comparisons were adjusted using the Bonferroni–Holm correction.

## 3. Results

Fourteen horses with unilateral forelimb lameness fulfilled the inclusion criteria. Horse descriptions are given in Table 1. Results of the lameness examination and diagnostic anaesthesia are shown in Table 2. On average, the 14 orthopaedic patients showed a mean lameness score of 2.4 ± 0.7/5 in a straight line on hard ground. Altogether, 10 perineural anaesthesia at the distal limb, 3 intrasynovial anaesthesia and 1 regional infiltration of the medial collateral ligament of the elbow joint with local anaesthetic were performed. Four cases were sound after regional or intrasynovial anaesthesia, seven cases showed a substantial reduction in lameness, lameness improved in one case and horses developed contralateral limb lameness following diagnostic anaesthesia in two cases (Table 2).

### 3.1. ANCOVA

The statistical analysis showed a significant interaction between the factors limb and anaesthesia for all phases of stance. The body weight showed a significant impact on the dependent variables (see Table 3). Due to the results of the ANCOVA, pairwise comparisons were made.

### 3.2. Pairwise Comparisons

#### 3.2.1. Before Diagnostic Anaesthesia

Before diagnostic anaesthesia, vertical force was significantly reduced on the lame limb compared to the sound limb during landing (−25%, *p* < 0.001), midstance (−20%, *p* < 0.001), and breakover (−27%, *p* < 0.001) (Table 4).

#### 3.2.2. After Diagnostic Anaesthesia

After diagnostic anaesthesia, the bilateral vertical force distribution became more symmetrical. Still, the horses continued to exert less vertical force in kg on the anaesthetised limb during landing (−6%, *p* = 0.1) and midstance (−3%, *p* = 0.3), whereas during breakover, the horses exerted more vertical force on the anaesthetised limb when compared to the sound limb (+5 %, *p* = 0.3). This bilateral difference in vertical force distribution was not significant in any motion event (Table 5).

#### 3.2.3. Lame Limb before and after Anaesthesia

The vertical force on the lame limb increased significantly after positive anaesthesia during all motion events (landing +15%, *p* = 0.009; midstance +16%, *p* < 0.001). The main increase in vertical force after anaesthesia was observed during breakover (+27%, *p* = 0.001) (Table 6).

#### 3.2.4. Sound Limb before and after Anaesthesia

During all motion events of the stance phase, the vertical force on the sound limb decreased, corresponding to the increase in vertical force on the anaesthetised limb. The reduction was not significant during landing (−8%, *p* = 0.06) and midstance (−4%, *p* = 0.1), whereas a significant vertical force reduction was again observed during breakover (−13%, *p* = 0.02) (Table 7).

## 4. Discussion

To our knowledge, this study is the first to show that after positive diagnostic anaesthesia of a lame limb, the main increase in vertical force on the lame limb and the main decrease in vertical force on the sound limb occur mainly during the breakover phase.

The results of this study confirmed our first hypothesis that before diagnostic anaesthesia, significant differences in vertical force between lame and sound limbs are detectable with the Hoof™ System. At a trot, a significant vertical force reduction on the lame limb compared to the sound limb occurred during landing, midstance, and breakover. This would be expected, as it has been documented by previous studies [12,13,14,17]. Additionally, the biggest difference in vertical force between lame and sound limbs occurred during breakover, followed by landing, which leads to the speculation that bilateral vertical force in these two phases is more sensitive to the presence of pain than the vertical force acting during midstance phase, which displayed a smaller difference between sound and lame limbs. It has been assumed that during landing, high forces affect the limb as impact peaks, shock, and vibration [27] probably accentuate lameness caused by articular or osseous disorders and energy storing tendons, e.g., the superficial digital flexor tendon and the suspensory ligament [28]. In contrast, during breakover, high forces are transferred to the ground for limb propulsion immediately before the swing phase; in particular, tendons such as the deep digital flexor tendon and muscles and ligaments such as the accessory ligament of the deep digital flexor tendon are subject to high strain [29,30,31], so pain associated with lesions of these structures may become more obvious. Further studies are warranted to investigate whether changes in the stance phase vary depending on the nature of the painful structure. Another potential cause is that vertical force on the sound limb is increased during breakover in order to propulse the horse, reducing the vertical force on the lame limb during the following stance phase [12]. In return, the vertical force on the lame limb is decreased during breakover because of pain and in order to lower the head during the following stance phase of the sound limb. Subsequently, the difference in vertical force between both forelimbs is greatest during breakover. During midstance, there is pure vertical loading with no acceleration, thus reducing microvibrations and instabilities associated with landing or breakover [32]. This may explain our findings and a lesser contribution of the midstance phase to lameness. These findings need to be further investigated as the Hoof™ System allows discrimination between phases of locomotion, which may be further considered for lameness management strategies. The differentiation of which part of the stance phase is mostly affected may also complement the output of trunk-mounted IMU sensor-based lameness detection systems, which are incapable of such discrimination, as they only perform a comparison of motion symmetry into impact and push-off lameness representing the first and second half of the stance phase [33].

The second hypothesis referred to the changes seen after diagnostic anaesthesia and can also be supported by the results of this study since following diagnostic anaesthesia, the vertical force affecting the anaesthetised limb increased significantly, whereas the vertical force on the sound limb decreased, which led to a more symmetrical bilateral vertical force during all parts of the stance phase. This was expected, as a more symmetric movement resulting from a more even loading between lame and sound limbs occurs following positive diagnostic anaesthesia, as described by a previous study [17]. The Hoof™ System could detect and calculate these changes numerically, which makes the system potentially useful to monitor the effect of diagnostic anaesthesia objectively in a clinical setting.

Our final hypothesis stating that the maximum effect of diagnostic anaesthesia occurs during midstance has to be rejected, as we observed the maximum increase in vertical force on the anaesthetised limb during breakover. This result emphasises the meaning of this phase for equine locomotion and compensation mechanisms of painful processes in the limb [12,20,34] so that the loss of sensory feedback after diagnostic anaesthesia [35] might become more obvious during this part of the stance phase. 

The observation that loading of front limbs was not completely symmetric after diagnostic anaesthesia was probably due to the different underlying pathologies inevitably leading to a variable improvement in lameness after diagnostic anaesthesia. In addition, it has been shown by pressure measuring that even sound horses may show an asymmetric bilateral loading [36]. This has to be considered in horses with mild lameness or those not responding to diagnostic anaesthesia. Theoretically, subtle hindlimb lameness becoming more dominant after positive anaesthesia of a lame front limb may also have influenced front limb loading through mechanisms of compensation [37]. To date, no threshold value for lameness has been found for the pressure mapping system used in the current study, as it has been described for body-mounted inertial sensor systems [9]. This will be necessary to support its routine clinical use. In a former study, it was reported that the Hoof™ System is not reliable compared to a force platform when being fixed with an equine hoof boot [22]. The reliability of mounting the sensor foils with adhesive tape was not evaluated in the current or former studies [18,19,21,34], which may have had an influence on the results. Another study could show reliability within but not between sessions when the sensor foils were attached with a glue-on shoe [38]. A potential cause for missing reliability between sessions could be sensel damage due to creases and delamination. Signs of disintegration, as described above, could also be observed in the current study, as measurements were performed on concrete flooring so that foam rubber and sensor foils were subject to abrasion, leading to the replacement of the sensors. To avoid the effect of humidity on sensor output, measurements took only place during dry weather [39]. 

In the current study, naturally lame horses were included, as we intended to investigate the system’s performance under real clinical circumstances. In contrast to an experimental model, this study design inevitably led to several limitations. The variation in lameness degree between individuals may have influenced the results and may be responsible for the large standard deviation seen in our samples. It is possible that individuals with higher degrees of lameness had the highest impact on our results. Further investigations regarding the effect of the degree of lameness on the changes in the different parts of the stance phase will be of interest. Whether abnormal gait patterns caused by disorders of the musculoskeletal system not diagnosed in the current study would lead to other deviations from normal gait patterns during the different parts of the stance phase will have to be proved by including more cases classified by specific disorders in future studies. As described above, our standardised examination protocols concerned the position of the handler, timings following diagnostic anaesthesia, and the speed of horses. While the first two were easily controlled, we did not measure the speed of horses, which may have influenced the results, as time, force, and spatial parameters are velocity-dependent [40]. At higher speed, more horses with subtle lameness are assessed as sound during subjective lameness evaluation, whereas more prominent lameness becomes more visible with higher speed [41,42]. While sound horses likely trot at a constant speed [43], lame horses tend to increase their speed after diagnostic anaesthesia [42]. At the same time, peak vertical force increases with increasing velocities [40]. This might have contributed, along with the analgetic effect of the local anaesthetic [23], to the significant increase in vertical force on the lame limb after anaesthesia. On the other hand, this is a potential reason why the vertical force reduction on the sound limb after diagnostic anaesthesia is not significant. The influence of the handler position was considered low, as in a previous study on sound horses, the handler position did not affect limb loading and hoof balance [15].

## 5. Conclusions

Diagnostic anaesthesia eliminates the vertical force distribution asymmetries between both forelimbs seen in unilaterally lame horses as evaluated by pressure measurements. In lame horses, differences in vertical force distribution found between the components of the stance phase contribute to a better understanding of equine gait and facilitate customised shoeing solutions, with breakover being the most affected. Hoof™ System measurements can be used to evaluate symmetric loading at a trot and to evaluate the effect of diagnostic anaesthesia in a clinical setting. Further studies including a larger number of horses with specific disorders are warranted to confirm the results of the current study.

## Figures and Tables

**Table 1:** Signalment of horses (age, weight, shod/unshod) with forelimb lameness included in the current study, divided by breed.

Breed	Age (Mean ± SD)	Weight (Mean ± SD)	Shod	Unshod
8 German Warmblood horses	12 ± 6 years	560 ± 50 kg	4 *	4
2 Icelandic horses	10 ± 5 years	350 ± 50 kg	0	2
2 Cold bloods	15 ± 2 years	650 ± 50 kg	2 ^†^	0
1 Appaloosa	16 years	500 kg	1 ^†^	0
1 Arabian horse	22 years	400 kg	0	1

* 2 × standard shoes, 1 × spider plate shoe, 1 × Eggbar shoe, ^†^ normal shoes.

**Table 2:** Results of lameness examination of horses with forelimb lameness included in the current study involving the breed, the lame forelimb, and the lameness score as well as location, type, and result of anaesthesia.

	Lame Limb	Lameness Score	Location of Anaesthesia	Type of Anaesthesia	Result of Anaesthesia
Warmbloods					
1	RF	2	antebrachiocarpal joint	intrasynovial	positive
2	LF	2	digital flexor tendon sheath	intrasynovial	positive with slight rest
3	RF	3.5	abaxial sesamoid	perineural	positive with lameness on the contralateral limb
4	RF	3	abaxial sesamoid	perineural	positive with slight rest
5	RF	2	abaxial sesamoid	perineural	positive with slight rest
6	LF	2	abaxial sesamoid	perineural	positive
7	RF	2	abaxial sesamoid	perineural	positive
8	RF	3	medial collateral ligament (elbow joint)	infiltration	positive with distinct rest
Icelandic horses					
9	LF	1	high 4 point	perineural	positive with slight rest
10	LF	2	low palmar digital	perineural	positive
Cold bloods					
11	RF	2	high palmar digital	perineural	positive with lameness on the contralateral limb
12	RF	3	low 4 point	perineural	positive with slight rest
Appaloosa					
13	RF	2	abaxial sesamoid	perineural	positive with slight rest
Arabian horse					
14	RF	3.5	antebrachiocarpal joint	intrasynovial	positive with slight rest

**Table 3:** Results of the ANCOVA. Global *p*-values for the repeated measurements limb (lame/sound) and anaesthesia (before/after), as well as their interaction and the covariable body weight during landing, midstance, and breakover.

	Landing	Midstance	Breakover
Impact of body weight	*p* = 0.001	*p* < 0.001	*p* = 0.02
Impact of limb (lame/sound)	*p* = 0.008	*p* = 0.003	*p* = 0.02
Impact of anaesthesia (before/after)	*p* = 0.6	*p* = 0.2	*p* = 0.5
Interaction between limb and anaesthesia	*p* = 0.003	*p* < 0.001	*p* < 0.001

**Table 4:** Pairwise comparisons of the bilateral vertical force in kg between the sound and the lame limb before anaesthesia during landing, midstance, and breakover.

	Lame Limb	Sound Limb	*p*-Value
Landing	111 ± 28 kg	147 ± 45 kg	<0.001
Midstance	265 ± 75 kg	332 ± 110 kg	<0.001
Breakover	135 ± 71 kg	185 ± 87 kg	<0.001

**Table 5:** Pairwise comparisons of the bilateral vertical force in kg between the sound and the lame limb after anaesthesia during landing, midstance, and breakover.

	Lame Limb	Sound Limb	*p*-Value
Landing	127 ± 34 kg	136 ± 35 kg	0.1
Midstance	307 ± 76 kg	318 ± 79 kg	0.3
Breakover	170 ± 73 kg	162 ± 63 kg	0.3

**Table 6:** Pairwise comparisons of the vertical force in kg on the lame limb before and after anaesthesia during landing, midstance, and breakover.

	Lame Limb: Vertical Force before Anaesthesia	Lame Limb: Vertical Force after Anaesthesia	*p*-Value
Landing	111 ± 28 kg	127 ± 34 kg	0.009
Midstance	265 ± 75 kg	307 ± 76 kg	<0.001
Breakover	135 ± 71 kg	170 ± 73 kg	0.001

**Table 7:** Pairwise comparisons of the vertical force in kg on the sound limb before and after anaesthesia during landing, midstance, and breakover.

	Sound Limb: Vertical Force before Anaesthesia	Sound Limb: Vertical Force after Anaesthesia	*p*-Value
Landing	147 ± 45 kg	136 ± 35 kg	0.06
Midstance	332 ± 110 kg	318 ± 79 kg	0.1
Breakover	185 ± 87 kg	162 ± 63 kg	0.02

## Data Availability

The data presented in this study are available on request from the corresponding author.
